# Evaluation of behavioural and neurochemical effects of psilocybin in mice subjected to chronic unpredictable mild stress

**DOI:** 10.1038/s41398-025-03421-4

**Published:** 2025-06-14

**Authors:** Ines Erkizia-Santamaría, Igor Horrillo, Nerea Martínez-Álvarez, Daniel Pérez-Martínez, Guadalupe Rivero, Amaia M. Erdozain, J. Javier Meana, Jorge E. Ortega

**Affiliations:** 1 Department of Pharmacology, University of the Basque Country UPV/EHU, Leioa, Bizkaia Spain; 2 Centro de Investigación Biomédica en Red de Salud Mental, Instituto de Salud Carlos III, Leioa, Spain; 3 Biobizkaia Health Research Institute, Barakaldo, Bizkaia Spain

**Keywords:** Depression, Pharmacodynamics

## Abstract

Depression and anxiety are disabling and high incidence mental disorders characterized by phenotypic heterogeneity. Currently available treatments show severe limitations. Thus, there is an urgent need for effective treatments in this population. In the search for novel rapid-acting antidepressants, the psychedelic psilocybin has emerged as a promising therapy in several clinical trials. However, its antidepressant mechanism of action is still not well understood. The aim of the present study was to evaluate the therapeutic potential of psilocybin in ameliorating the adverse behavioural and neurochemical consequences of chronic stress. To this end, a chronic unpredictable mild stress (CUMS) animal model was used, and psilocybin treatment was administered (two doses of 1 mg/kg, i.p., administered 7 days apart). Psilocybin reversed impairments in anhedonia and behavioural despair dimensions of depressive phenotype but not in apathy-related behaviour. Psilocybin administration was also able to exert an anxiolytic-like effect on treated animals. Physiological alterations caused by stress, indicative of a hyperactive hypothalamic-pituitary-adrenal axis (HPA), were not reversed by psilocybin. When neuroplasticity-related proteins were assessed in cerebral cortex, brain-derived neurotrophic factor (BDNF) was found to be decreased in stressed animals, and treatment did not reverse such impairment. Psilocybin administration increased the expression and function of serotonin-2A-receptor (5HT2AR) in brain cortex of control and CUMS groups. Furthermore, psilocybin treatment caused a selective increase in the expression of glucocorticoid-receptor (GR) in brain cortex of CUMS mice. In conclusion, psilocybin was able to rescue impairments in the depressive phenotype, and to induce anxiolytic-like effects. Furthermore, an enhancement in sensitivity to psilocybin-induced HTR was observed following a booster dose. Altogether, this work provides new knowledge on the putative benefit/risk actions of psilocybin and contributes to the understanding of the therapeutic mechanism of action of psychedelics.

## Introduction

Major depressive disorder (MDD or depression) is one of the leading causes of burden of disease worldwide [1, 2]. Anxiety is common in the context of depression and approximately two-thirds of individuals diagnosed with MDD also experience clinical anxiety [1]. While numerous treatments for MDD are available, many patients do not respond adequately to traditional antidepressants. One third to half of patients do not respond to multiple treatment steps, and more might only obtain a partial response [3]. This consequently prolongs the functional burden associated with MDD, with negative consequences on occupation, social relationships and physical health, among others [4]. Hence, there is a compelling need to develop new treatment strategies for MDD.

Currently, there is increasing evidence suggesting that serotonergic psychedelics, particularly psilocybin, behave as fast-acting and long-lasting therapeutic agents. According to various clinical trials, psilocybin improves symptoms associated to affective disorders after single or double exposure to the drug [5–10]. Psilocybin can also reduce morbidity in patients with various forms of anxiety disorders [11] and has shown promising effects on depression and anxiety in people with terminal illnesses (for review see [12]). Psilocin, the dephosphorylated active form of psilocybin, presents high affinity for the serotonin 2A receptor (5HT2AR), the main mediator of the acute psychedelic effects caused by the drug in humans [13]. Nonetheless, the pharmacology of psilocin is complex. It has been reported that psilocybin/psilocin can exert their effects through the activation of several serotonergic receptors [14] and also through high-affinity binding to tropomyosin receptor kinase B receptors (TrkB), the receptor for brain-derived neurotrophic factor (BDNF) [15]. In consequence, there is an imperative need to gain mechanistic understanding of the therapeutic effects of psilocybin to ultimately implement effective and feasible treatment models of psychedelic-assisted therapies for depression-associated symptomatology.

Despite MDD’s multifactorial nature, which encompasses genetic, biochemical, psychosocial and environmental factors, exposure to chronic stress in known to be a major precipitant for its development in humans. Extensive evidence supports the link between chronic stress, hypothalamic-pituitary-adrenal axis (HPA) dysfunction and depressive disorders [16, 17]. HPA impairment negatively affects different brain areas involved in mood regulation, such as the prefrontal cortex (PFC) [18]. In agreement, multiple imaging studies have confirmed both structural and functional disturbances in the brains of MDD patients, some of which are modulated by treatment with antidepressants [19].

In order to better comprehend the neurobiological mechanisms of depression and treatment efficacy, different translational animal models have been developed [20, 21]. In the context of MDD, notable attention is drawn to the chronic unpredictable mild stress model (CUMS). This model has been described as a valid, reliable and useful tool for the study of the neurobiological basis of depression [22]. CUMS animal model has demonstrated construct validity and is able to reproduce neurobiological alterations observed in MDD patients [23]. In consequence, it has been suggested that the model may be a useful proxy for the identification of novel impaired targets in depression and anxiety-like states [24]. Interestingly, CUMS offers good predictive validity, showing specific and selective responsiveness to clinically effective antidepressants [25]. In this regard, several pre-clinical trials have evaluated antidepressant properties of psilocybin, either in naïve rodents [26–28] or in animal models of disease. Different studies have also focused on anxiolytic effects of psychedelics, including psilocybin (for review see [29]). In selectively bred rat strains, psilocybin shows conflicting results when testing antidepressant potential [30, 31]. Other works have reported long-lasting antidepressant or anxiolytic effect of psilocybin in corticosterone-induced anhedonia in mice [32] and in a repeated swimming stress model [33]. Nonetheless, to the best of our knowledge, only one work has evaluated the antidepressant effect of psilocybin in a chronic stress model in mice [34]. In this pioneering study that employed the chronic multimodal stress, single psilocybin administration acutely improved anhedonia-like behaviour in mice 24 h after drug administration. Unfortunately, long-lasting effects were not tested in the model.

Considering the growing clinical and public interest in psilocybin, there is a clear need for a comprehensive study on antidepressant- and anxiolytic-like effects of psilocybin through the use of an array of behavioural tests in a translational animal model of disease [35]. We propose that models with extensive stress exposure, such as CUMS paradigm (social and non-social stressor combination), may offer a more informative framework for the study of antidepressant- and anxiolytic-like effects of psychedelics. Previously, a dose-response study for acute psilocybin-induced head-twitch response (HTR, one of the best known translational assays to characterize the acute effects of psychedelics in rodents) was performed in order to support dose finding for psilocybin long-lasting effects evaluation [14]. Nevertheless, the role of psychedelic-induced subjective effects in therapeutic outcomes is still debated [36]. Against this background, the aim of the present study was to conduct a wide behavioural characterization of psilocybin treatment (two doses of 1 mg/kg, i.p., 7 days apart) in a CUMS animal model through specific tests for hallucinogenic-, depression- and anxiety-like phenotypes and evaluate the therapeutic potential of psilocybin in ameliorating the adverse consequences of chronic stress. In addition, the role of HPA, 5HT2AR and neuroplastic effects behind therapeutic effects of psilocybin were assessed.

## Materials and methods

### Animals

Adult male C57BL/6J mice (8 weeks old) were purchased from Envigo (Barcelona, Spain) and housed under standard laboratory conditions on a 12 h light/dark cycle, at room temperature (22–24 °C), with food and water available *ad libitum*. The animal care and experimental protocols were carried out in accordance with the principles of animal care established by the EU Directive 2010/63/EU and in agreement with Spanish legislation (Royal Decree 53/2013), and were approved by the UPV/EHU Ethical Board of Animal Welfare (CEEA; reference M20_2020_014), as well as in compliance with ARRIVE guidelines [37].

### Chronic unpredictable mild stress (CUMS)

**Fig. 1:**
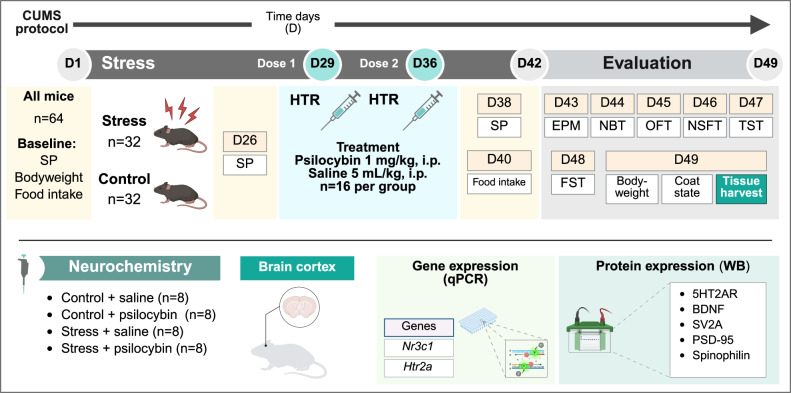
Schematic representation of experimental design. Sucrose preference (SP), head-twitch response (HTR), elevated plus maze (EPM), nest-building test (NBT), open field test (OFT), novelty-suppressed feeding test (NSFT), tail-suspension test (TST), forced swimming test (FST), glucocorticoid-receptor gene (*Nr3c1*), serotonin 2A receptor gene (*Htr2a*), serotonin 2A receptor (5HT2AR), brain-derived neurotrophic factor (BDNF), synaptic vesicle glycoprotein 2A (SV2A), postsynaptic density protein 95 (PSD-95). Created with biorender.com.

Thirty-two mice were randomly assigned to the CUMS group and 32 to the control group (Fig. 1). Stressed animals were housed individually. The stress paradigm was designed based on previously described protocols, with minor modifications [38–40]. The duration of the stress protocol was 6 weeks (42 days of stress), where daily stressors were applied in a random manner. Three to four different stimuli were applied every day, and were not repeated on consecutive days. A combination of stressors was designed to reach an equilibrium of stress intensity every day of the protocol, according to a severity point system (1: mild, 2: moderate, 3: high) [41]. Stressors were combined to reach 8–11 points each day during the stress protocol (Table S1).

### Drugs and treatment

Psilocybin [3-[2-(dimethylamino)-ethyl]-1H-indol-4-ol dihydrogen phosphate] was obtained from THC Pharm (Frankfurt, Germany) and was dissolved to 0.2 mg/mL in saline solution (0.9% NaCl).

Two doses of psilocybin (1 mg/kg, i.p.) or saline (5 mL/kg, i.p.) were administered on the last two weeks of the stress protocol: week 5 (day 29) and week 6 (day 36) (Fig. 1). The experimental groups were control-saline; control-psilocybin; CUMS-saline; CUMS-psilocybin (*n* = 16 per group for behavioural evaluation, of which *n* = 8 per group for in vitro assessments). The rationale for the treatment design was to mimic the posology of various clinical trials in which patients received two high (hallucinogenic) doses of psilocybin [5–7]. The 1 mg/kg dose of psilocybin was previously characterized as the most potent HTR-inducing dose in mice [14]. This dose was selected to evaluate long-term antidepressant and anxiolytic effects in the animal model.

### Behavioural evaluation

Immediately following stress protocol cessation, animals were subjected to a battery of behavioural tests in order to evaluate the depressive- and anxiety-like phenotype of stressed animals and the potential reversion induced by psilocybin treatment [24] (Fig. 1). The tests were carefully selected on the basis of utility, frequency of use in previous publications in the assessment of depression-like and anxiety-like behaviours and the use of different tests that will support each other when necessary [42]. All of the animals, without exception, were subjected to the full behavioural battery. The order of assays was established according to recommendations, starting with the tasks requiring maximal effects of novelty and ending with the most stressful, as previously recommended [38]. This strategy reduces the impact of the behavioural tests previously carried out during a behavioural battery protocol. We have included a broader battery of behavioural tests in order to draw a complete assessment and avoiding reaching conclusions based on the quantification of a single test. The full series of behavioural tests was completed by all four groups of animals (control-saline; control-psilocybin; CUMS-saline; CUMS-psilocybin). A meticulous approach was adopted when conducting comparisons between the different groups and the control-saline group. This was done in order to ensure that the influence of cumulative stress exerted during the consecutive behavioural tests was controlled. The protocol was also conceived with the objective of ensuring, as far as possible, that any neurochemical changes observed in the in vitro tests were induced by the CUMS protocol or the pharmacological treatment, and not by the stress associated with the different behavioural tests performed.

### Head-twitch response (HTR)

Immediately following psilocybin/saline administration, animals were placed in an open field arena (43 × 43 × 43 cm) (60 lux). They were recorded for 25 min. A trained and blinded observer manually quantified the HTR for the last 20 min [14]. The HTR was measured in psilocybin-treated animals (*n* = 16). However, since saline-treated mice exhibited an HTR close to zero in every single animal, only a subgroup of animals (*n* = 8) was required for the control-saline and CUMS-saline groups evaluation.

### Sucrose preference (SP)

To determine the effects on anhedonia-like behaviour, the SP was performed as previously described [43], with minor modifications. An 8-h fasting period was set to promote water/sucrose (2%) consumption. Bottles were placed in cages at 17:00, and left for 15 h. Previous to the beginning of the stress protocol, a training phase was performed for 48 h in all groups of animals, in which bottle positions were switched every 24 h in order to avoid place preference. The hedonic state of the animals was evaluated individually at repeated time-points during experimental protocol: baseline (week 0, day −2), week 4 (day 26, before psilocybin administration) and week 6 (day 38, after psilocybin administration). CUMS mice were not subjected to stress during the test. After SP test, animals were returned to the stress schedule as established. Sucrose preference was calculated as percentage (sucrose consumption - water consumption / sucrose consumption + water consumption x 100) [32].

### Elevated plus maze (EPM)

EPM testing was performed on day 43 as previously described [44]. The time in open arms was evaluated in the video tapes by a blinded, trained researcher. The percentage of entries to open arms was calculated as entries to open arms / total amount of entries to open and closed arms x 100.

### Nest-building test (NBT)

The spontaneous motivation of the animals was measured on the NBT, on day 44. Each mouse was kept in individual cages. One square of nesting material was introduced in each cage and mice were left undisturbed to build the nest. Nest-building skills were evaluated 30 min later. The nest quality was evaluated according to a scale using the following criteria: 1 (cotton square is intact), 2 (cotton square is partially used), 3 (cotton is scattered, but there is no form of nest), 4 (cotton is gathered but there is a flat nest), 5 (cotton is gathered to a ball-shaped nest) [45].

### Open field test (OFT)

The OFT has been used to measure locomotor activity and emotionality from exploration and anxiety in rodents [46]. The OFT was performed on day 45. Mice were carefully placed in the centre of the arena and left to explore for 10 min [47]. Videos were analysed using automated tracking software Smart 3.0 (PanLab SL, Barcelona, Spain). The total distance (cm) and time spent in the centre of the arena (s) were evaluated.

### Novelty-suppressed feeding test (NSFT)

To further test anxiety-like behaviour, the NSFT was performed [48] on day 46. Food pellets were removed from cage grids 16 h prior to the NSFT. An open field arena was filled with sawdust and a single pellet attached to the ground was placed in the centre. The room was kept dark during the whole duration of the test except for a light bulb that illuminated the pellet (800–900 lux). Chewing or biting the pellet was established as the criterion to set the latency and remove the animal from the arena. A period of 10 min was set as the maximum latency time permitted.

### Tail suspension test (TST)

The behavioural despair was evaluated using a TST apparatus (PanLab SL, Barcelona, Spain) on day 47. Mice were hung from a piece of tape stuck to the tail. The duration of the test was 6 min [49], and the immobility time was manually evaluated by tape visualisation for the whole duration.

### Forced swimming test (FST)

The modified Porsolt swim test was carried out as previously described [50, 51], on day 48. Four cylinders (24 cm height × 20 cm diameter) were filled with water (24 ± 1 °C, 18 cm height). The mouse was determined to be immobile when floating in an upright position without other activity than the necessary to keep its head above water. Mice were judged to be swimming if they were making active movements (usually horizontal) throughout the chamber. The climbing behaviour was defined as upward directed movements with the forepaws along the wall of the chamber. The mobility (swimming and climbing behaviours) and the immobility were quantified during the last 4 min of the test, as previously described [52].

### Coat state

An assessment of the coat state was completed as a measure of decreased grooming and disturbed self-directed behaviour [45]. On day 49, coating was evaluated in the head, neck, back and abdomen, and a score was given to each body part: 0, good state (fur is shiny and smooth, no tousled or spiky patches); 0.5, moderately bad state (fur is slightly fluffy with some spiky patches); 1, bad state (fur is dirty and fluffy). The total score was calculated as the sum of such body parts.

### Physiological state evaluation

#### Bodyweight

All mice were weighed before stress protocol, then randomly assigned to control or CUMS groups. Subsequently, mice were weighed before euthanasia. The bodyweight gain relative to baseline (%) was reported as a measure of a physical sign of chronic stress.

#### Food intake

Prior to the beginning of stress (baseline) and during the 5th week (day 40–41) of the stress protocol, the food intake was evaluated in all experimental groups. CUMS mice were subjected to a stimulus that does not hinder food consumption (white noise). Pellets in each cage were weighed at 17:00, and mice were left to consume food and water *ad libitum* overnight until 11:00 the following day. The consumed amount of food was corrected for bodyweight (g food/g bodyweight).

#### Tissue harvesting

On day 49 (7 days post-stress cessation and 14 days after last psilocybin dose), mice were euthanized through cervical dislocation, brains were quickly removed; whole cortices were extracted and immediately frozen. Peripheral tissues were dissected to obtain white adipose tissue (WAT), brown adipose tissue (BAT) and adrenal glands. Tissues were weighed immediately after dissection.

### Calculations of z-scores for behavioural and physiological assessments

The z-scoring technique allows the integration of a battery of behavioural tests that evaluate similar phenotypes in mice [53, 54]. Z-scores (*z*) are dimensionless mathematical tools (standard scores) that provide a mean-normalization of continuous variables in order to compare related data across studies [55]. It was calculated as follows: z = (x - µ) / σ, where z = z score; *x* = value of observed parameter; *μ* = mean of control group and *σ* = SD of control group. Combination of various z-scores allows to assess overall symptom severity.

Z-scores of individual parameters measured in each behavioural test were calculated. Then, integrated parameters were obtained by combining such scores within test. Subsequently, integrated z-scores were again combined to calculate scores for symptomatic dimensions within the depressive phenotype, and finally an overall score for each phenotype was obtained: depression index, anxiety index and index of physiological signs of stress.

This methodology was applied to study the overall behavioural effects caused by stress and/or psilocybin treatment. The control-saline group was defined as the control group for parameters *μ* and *σ*. The directionality of scores was adjusted so that increased score values reflected increased dimensionality. For the calculation of the depression index anhedonic behaviour (SP) and behavioural despair (TST, FST) were normalised. Categorical ordinal variables (NBT, coat state) were not taken into consideration in the calculation of the depression index, since normalisation of non-continuous variables is meaningless. For the calculation of the anxiety index, data from the EPM, NSFT and OFT were used. Finally, for physiological signs of stress the bodyweight gain, food intake and adrenal gland weight were normalised.

### Real-time quantitative PCR (qPCR)

Brain cortices were used for RNA extraction (*n* = 8 per group) as previously described [56]. qPCR was performed in cDNA with a StepOne System (Applied Biosystems, Foster city, CA, USA) using TaqMan gene expression assays for glucocorticoid receptor *Nr3c1* (interrogated sequence NM_008173.3, assay ID Mm00433832_m1), and for *Htr2a* (interrogated sequence NM_172812.2, assay ID Mm00555764_m1). All samples were run in triplicates. mRNA expression of target genes was corrected with that of reference genes *Rps29* (ribosomal protein S29) and *ActB* (actin beta), and with a reference sample (pool of all samples) using ΔΔCt method: ΔΔCt = (Ct (target gene)_sample_ – mean Ct (reference genes)_sample_) – (Ct (target gene)_pool_ – mean Ct (reference genes)_pool_). The relative amount of mRNA was calculated as 2^−ΔΔCt^.

### Western blot

Samples were prepared as previously described [57], with minor modifications. Brain cortices were processed as total homogenates for Western blot experiments (*n* = 8 per group). Samples were prepared in electrophoresis buffer. Denatured proteins (40 µg) were resolved in SDS-PAGE gels and transferred to nitrocellulose membranes. After being blocked for 1 h at room temperature (3% non-fat powdered milk in PBS), membranes were incubated overnight at 4 °C under constant agitation with the primary antibodies against 5HT2AR (Immunostar 24288, 1:500; Immunostar, Hudson, WI, USA); BDNF (Ab108319, 1:2000; Abcam, Cambridge, UK); synaptic vesicle glycoprotein 2A (SV2A) (Ab32942, 1:1000; Abcam, Cambridge, UK); postsynaptic density protein 95 (PSD-95) (MAB1596, 1:2000; Merck Millipore, Darmstadt, Germany); spinophilin (PA5-48102, 1:1000; ThermoFisher/Invitrogen, Carlsbad, CA, USA) or β-actin (A1978, 1:200,000; Sigma-Aldrich, Burlington, MA, USA), as a loading control. After incubation with the chemiluminescent secondary antibodies (anti-rabbit, HRP-conjugated, Jackson ImmunoResearch 111-035-144, 1:5000; anti-mouse, HRP-conjugated, Jackson ImmunoResearch 115-035-146, 1:5000; Jackson ImmunoResearch, West Grove, PA, USA; anti-sheep, HRP-conjugated, A16041, 1:4000; ThermoFisher, Carlsbad, CA, USA) for 1–2 h at room temperature, immunotransference substrate (ECL Pierce^TM^, Thermofisher; Carlsbad, CA, USA) was added and the immunoreactive signal (integrated intensity value) was detected using the Amersham Imager 680 (Cytiva, Little Chalfont, Buckinghamshire, UK) and quantified using Image Studio Lite 5.2 (LI-COR Biociences, Lincoln, NE, USA). A standard pool of total homogenate was processed in the same gels and used as external reference sample. Immunoreactivity values of each target protein were normalized for β-actin signal of the same sample. Whole uncropped images of the original Western blots from which figures have been obtained are shown in Figure S1.

### Statistical analysis

All experiments were randomized and analysed in blind. The required sample sizes were estimated on the basis of our past experience performing similar experiments and power analysis calculations performed with GPower 3.1.9.7 software (University of Düsseldorf, Düsseldorf, Germany). Data were tested initially for normality (Shapiro-Wilk) and further analysed. Behavioural, physiological, Western blot and qPCR experimental, data were analysed using two-way ANOVA followed by Bonferroni *post hoc* test after statistically significant interaction between factors. Factors were identified as F_CUMS_ (control vs stress condition), F_Psil_ (saline vs psilocybin treatment) and F_i_ (interaction between factors). For comparison of the CUMS effect before treatment and the HTR between first and second administration of psilocybin, data were analysed using paired *t*-test. Semi-quantitative parameters (coat state, NBT) were analysed using Kruskal-Wallis test (non-parametric ANOVA), followed by Dunn’s multiple comparisons test. All results are shown as mean ± SEM. In all cases, statistical significance was considered when *p* < 0.05. Statistical outliers were identified using the Grubbs’ test. Degrees of freedom are summarized in Table S2. Data were analysed using GraphPad Prism 10.0 (GraphPad Software, San Diego, CA, USA).

## Results

### Behavioural evaluation

#### Head-twitch response (HTR)

**Fig. 2:**
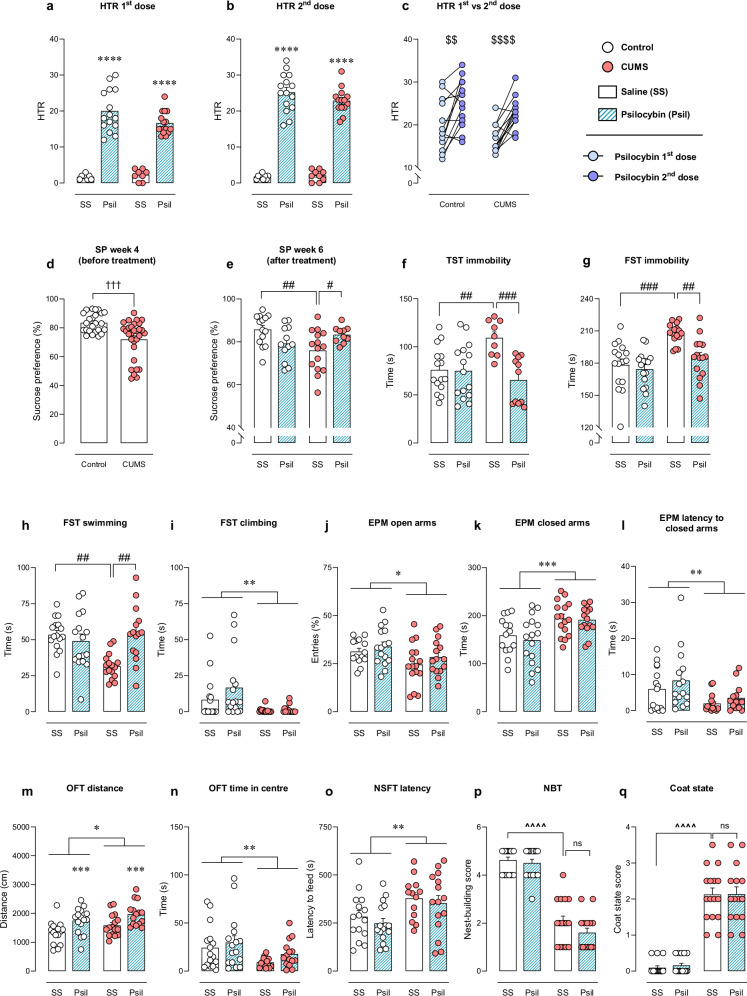
Behavioural evaluation. Evaluation of HTR in the first **a** and second administration **b** of psilocybin (1 mg/kg, i.p.) or saline, and comparison **c** of psilocybin-induced HTR between first and second dose. Evaluation of anhedonia in SP during stress **d** and during stress after treatment **e**. Immobility in TST **f**. Immobility **g**, swimming **h** and climbing **i** in FST. Percentage of entries to open arms **j**, time in closed arms **k** and latency to entrance to closed arms in EPM **l**. Travelled distance **m** and time in central square **n** in OFT. Latency to feed in NSFT **o**. Evaluation of performance in NBT **p**. Evaluation of coat state **q**. Data are presented in circles for individual quantification and in bars as mean ± SEM. Two-way ANOVA. *p < 0.05, **p < 0.01, ***p < 0.001, ****p < 0.0001. Bonferroni *post hoc* test. ^#^*p* < 0.05, ^##^*p* < 0.01, ^###^*p* < 0.001. Paired *t*-test. ^$$^*p* < 0.01, ^$$$$^*p* < 0.0001. Unpaired *t*-test. ^†††^*p* < 0.001. Dunn’s *post hoc t*est. ^^^^*p* < 0.0001.

The first dose of psilocybin (day 29) induced 20.00 ± 1.51 HT events in the control mice (*n* = 16) and 16.67 ± 0.82 HT events in the CUMS mice (*n* = 16). As expected, saline administration did not induce HTR in either control or CUMS groups (*n* = 8). A significant effect of psilocybin was revealed (F_Psil_(1,42) = 182.600, *p* < 0.0001). No significant effect of stress or interaction between factors was found (Fig. 2a). A more detailed statistical analysis is shown in table S3. The second administration of systemic psilocybin (day 36) induced 25.20 ± 1.34 HT events in the control and 22.86 ± 0.94 HT events in the CUMS mice. As expected, a significant effect of psilocybin was found (F_Psil_(1,43) = 405.200, *p* < 0.0001), but no significant effect of stress or significant interaction between factors was identified (Fig. 2b).

Interestingly, a significant difference was observed in the HTR between first and second administration of psilocybin, within treatment-groups. The control group exhibited a higher number of head-twitches on the second administration (t = 3.107, *p* < 0.01). Likewise, higher HTR was observed in the CUMS animals on the second psilocybin administration (t = 5.753, *p* < 0.0001) (Fig. 2c).

#### Sucrose preference test (SP)

As expected, no differences were found at baseline in the SP between groups (data not shown). Prior to the first dose of psilocybin/saline administration, stressed animals showed significant reduction in preference (t = 3.80, *p* < 0.001) (Fig. 2d). All numerical values are included in Table S4.

After drug administration, CUMS-psilocybin animals showed increased preference, compared to their saline-treated counterparts. Two-way ANOVA showed significant interaction between factors (F_i_(1,47) = 9.923, *p* < 0.01). *Post hoc* analysis revealed significant differences between control-saline and CUMS-saline (t = 3.373, *p* < 0.01), and between CUMS-saline and CUMS-psilocybin mice (t = 2.316, *p* < 0.05) (Fig. 2e).

#### Tail suspension test (TST)

The CUMS-saline group showed increased immobility-time in the TST. This effect was rescued by treatment in the CUMS group. Two-way ANOVA revealed significant effect of treatment and significant interaction between factors (F_Psil_(1,46) = 9.907, *p* < 0.01; F_i_(1,46) = 9.045, *p* < 0.01). *Post hoc* analysis showed differences between control-saline and CUMS-saline (t = 3.207, *p* < 0.01), and between CUMS-saline and CUMS-psilocybin (t = 3.965, *p* < 0.001) groups (Fig. 2f).

#### Forced swimming test (FST)

Increased immobility-time induced by CUMS was observed in the FST. Psilocybin rescued such effect in stressed mice (Fig. 2g). Two-way ANOVA revealed significant effect of CUMS, treatment and interaction between factors (F_CUMS_(1,59) = 19.740, *p* < 0.0001; F_Psil_(1,59) = 9.024, *p* < 0.01; F_i_(1,59) = 4.454, *p* < 0.05). The *post hoc* analysis showed differences between control-saline and CUMS-saline (t = 4.672, *p* = 0.0001), and between CUMS-saline and CUMS-psilocybin groups (t = 3.587, *p* < 0.01).

Such behavioural effects were reflected in alterations in swimming (F_CUMS_(1,59) = 4.738, *p* < 0.05; F_Psil_(1,59) = 5.235, *p* < 0.05; F_i_(1,59) = 11.67, *p* < 0.01) (Fig. 2h). *Post hoc* analysis showed differences between control-saline and CUMS-saline groups (t = 3.987, *p* = 0.01), and between CUMS-saline and CUMS-psilocybin groups (t = 4.001, *p* < 0.01). A decreased climbing was also observed in both CUMS groups, but psilocybin did not modify such behaviour (F_CUMS_(1,59) = 10.98, *p* < 0.01; F_Psil_(1,59) = 1.729, *p* = 0.194; F_i_(1,59) = 1.271, *p* = 0.264) (Fig. 2i).

#### Elevated plus maze (EPM)

The percentage of entries to open arms in EPM was reduced in stressed group (F_CUMS_(1,57) = 6.501, *p* < 0.05). No significant effect of psilocybin or interaction between factors was found (Fig. 2j). Likewise, stressed groups exhibited increased time in the closed arms (F_CUMS_(1,57) = 14.910, *p* < 0.001) (Fig. 2k). The latency to entrance to closed arms was significantly lower for CUMS mice compared to controls, as revealed by two-way ANOVA (F_CUMS_(1,55) = 9.37, *p* < 0.01). No significant effect of psilocybin or significant interaction between factors were found (F_Psil_(1,55) = 1.79, *p* > 0.05; Fi(1,55) = 0.08, *p* > 0.05) (Fig. 2l).

#### Open field test (OFT)

The CUMS protocol induced increased exploratory activity in the OFT, as well as psilocybin did (F_CUMS_(1,59) = 5.207, *p* < 0.05; F_Psil_(1,59) = 15.40, *p* < 0.001; F_i_(1,59) = 0.009, *p* = 0.921) (Fig. 2m). The time in centre was significantly reduced in CUMS groups regardless of treatment (F_CUMS_(1,58) = 7.383, *p* < 0.01) (Fig. 2n).

#### Novelty-suppressed feeding test (NSFT)

An increased latency to feed in the NSFT was observed in CUMS groups, and psilocybin did not rescue such increase (F_CUMS_(1,55) = 9.347, *p* < 0.01; F_Psil_(1,55) = 0.835, *p* = 0.365; F_i_(1,55) = 0.020, *p* = 0.889) (Fig. 2o).

#### Nest-building test (NBT)

The CUMS protocol caused deficits in the ability of mice to make nests, as decreased scores were obtained in the NBT, but psilocybin did not rescue such deficits (non-parametric ANOVA Kruskal-Wallis = 47.71, *p* < 0.0001). Dunn’s *post hoc* test showed significant differences between control-saline and CUMS-saline (Z = 4.64, *p* < 0.0001) groups, but no effect of psilocybin (CUMS-saline vs CUMS-psilocybin, Z = 0.76, *p* > 0.05) (Fig. 2p).

#### Coat state

CUMS animals showed decreased self-care behaviour, evidenced by degradation of the coat state (Kruskal-Wallis = 49.67, *p* < 0.0001; control-saline vs CUMS-saline, Z = 5.19, *p* < 0.0001; CUMS-saline vs CUMS-psilocybin, Z = 0.03, *p* > 0.05) (Fig. 2q).

### Physiological state evaluation

#### Bodyweight

**Fig. 3:**
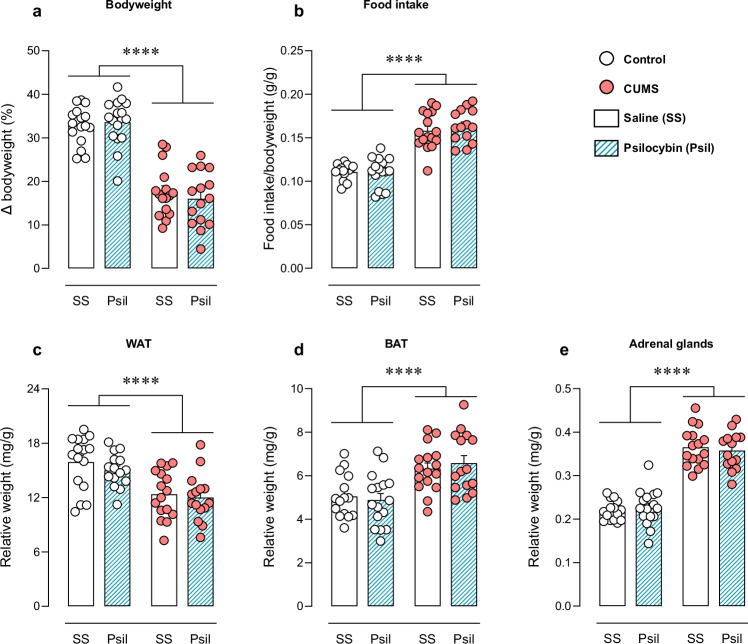
Physiological evaluation. Bodyweight gain relative to baseline weight (%) **a**, food intake normalized by bodyweight (g/g) **b**, weight relative to bodyweight of white adipose tissue (WAT) **c**, brown adipose tissue (BAT) **d** and adrenal glands **e** (mg/g). Data are presented in circles for individual quantification and in bars as mean ± SEM. Two-way ANOVA. ****p < 0.0001.

Baseline differences between groups were previously discarded (data not shown). At the end of stress protocol, CUMS animals showed lower bodyweight gain. Differences were observed for the CUMS factor (F_CUMS_(1,59) = 140.400, *p* < 0.0001) but not for the psilocybin factor or interaction between the two variables (Fig. 3a).

#### Food intake

Increased food intake on week 5 was observed in CUMS groups (F_CUMS_(1,58) = 134.200, *p* < 0.0001). Psilocybin treatment did not affect the food intake in control nor in CUMS mice (Fig. 3b).

#### Tissue weight

Stressed animals exhibited significantly lower WAT weight relative to bodyweight (F_CUMS_(1,58) = 25.430, *p* < 0.0001). No psilocybin effect or significant interaction was found (Fig. 3c). In contrast, stressed animals exhibited significantly higher BAT weight relative to bodyweight (F_CUMS_(1,58) = 25.030, *p* < 0.0001). Again, no psilocybin effect or significant interaction was found (Fig. 3d).

Consistent with the physiological effects of a chronic hyperactivation of HPA, CUMS animals showed increased adrenal weight relative to bodyweight (F_CUMS_(1,57) = 193.000, *p* < 0.0001). However, psilocybin treatment did not show any impact on CUMS-induced adrenal hypertrophy (Fig. 3e).

### Z-score for depression index, anxiety index and physiological signs of stress calculation

In order to carry out a comprehensive analysis of the behavioural impairments caused by chronic stress and the therapeutic effects induced by psilocybin treatment, z-scoring methodology was employed [53–55]. This transformation is essential for evaluating the effects of psilocybin in different domains of the disease modelled by the CUMS protocol, thus providing a comprehensive overview that goes beyond the interpretations made by individual tests. Individual parameters measured were normalized (Table S5), then integrated parameters were obtained by combining scores within test, to finally draw a z-score for each phenotype or physiological sign: depression index, anxiety index and physiological signs of stress (Figures S2, S3 and S4).

**Fig. 4:**
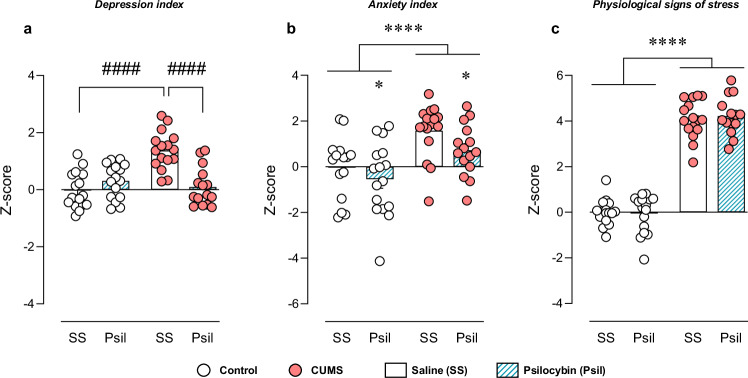
Z-Scores. Z-scores of *Depression index* **a***, Anxiety index* **b** and *Physiological signs of stress* **c**. Data are presented in circles for individual quantification and in bars as mean ± SEM. Two-way ANOVA. *p < 0.05, ****p < 0.0001. Bonferroni *post hoc* test. ^####^*p* < 0.0001.

Regarding depression index, two-way ANOVA revealed significant effect of stress, treatment and interaction between factors (F_CUMS_(1,59) = 13.18, *p* < 0.001; F_Psil_(1,59) = 8.42, *p* < 0.01; F_i_(1,59) = 24.27, *p* < 0.0001). *Post hoc* analysis showed differences between control-saline and CUMS-saline (t = 6.10, *p* < 0.0001), and between CUMS-saline and CUMS-psilocybin (t = 5.49, *p* < 0.0001) (Fig. 4a).

Anxiety index was also affected by stress (F_CUMS_(1,58) = 18.26, *p* < 0.0001). Interestingly, an anxiolytic effect after psilocybin administration was also observed (F_Psil_(1,58) = 4.02, *p* < 0.05). No significant interaction between factors was revealed (F_i_(1,58) = 0.27, *p* = 0.61) (Fig. 4b). Thus, global anxiolytic effect of psilocybin seems to be induced both in stressed and non-stressed mice.

Finally, overall effects on physiological signs of stress were analysed. Two-way ANOVA revealed significant effect of stress (F_CUMS_(1,58) = 446.7, *p* < 0.0001), but no effect of treatment (F_Psil_(1,58) = 0.05, *p* = 0.83) or interaction between factors (F_i_(1,58) = 0.25, *p* = 0.61) (Fig. 4c).

### *Nr3c1* and *Htr2a* mRNA expression in cortical samples

**Fig. 5:**
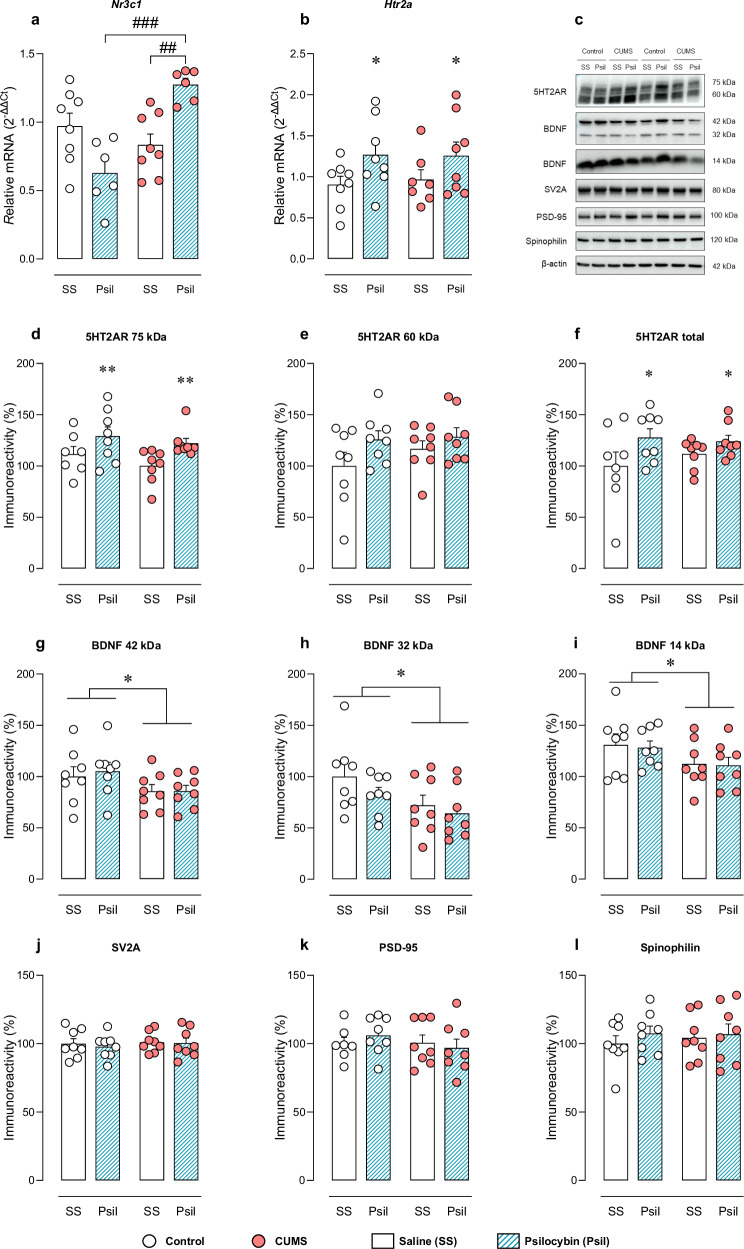
Neurochemical evaluation. Relative mRNA levels of *Htr2a* **a**, *Nr3c1* **b**. Representative Western blot of 5HT2AR, BDNF, SV2A, PSD-95 and spinophilin **c**. Relative protein expression of 5HT2AR (75 kDa, 60 kDa and total) **d**, **e**, **f**, BDNF (42 kDa, 32 kDa and 14 kDa) **g**, **h**, **i**, SV2A **j**, PSD-95 **k** and spinophilin **l** in brain cortex. Two-way ANOVA. *p < 0.05, **p < 0.01. Bonferroni *post hoc* test. ^##^*p* < 0.01, ^###^*p* < 0.001.

A selective increase in relative *Nr3c1* expression in CUMS-psilocybin group was found, (F_CUMS_(1,24) = 8.756, *p* < 0.01; F_Psil_(1,24) = 0.319, *p* = 0.577; F_i_(1,24) = 20.66, *p* < 0.001). P*ost hoc* analysis showed differences between control-psilocybin and CUMS-psilocybin groups (t = 4.964, *p* < 0.001), and between CUMS-saline and CUMS-psilocybin groups (t = 3.613, *p* < 0.01) (Fig. 5a).

Relative *Htr2a* mRNA expression was increased in both non-stressed and stressed psilocybin-treated groups (F_Psil_(1,27) = 5.553, *p* < 0.05). No significant effects were found for CUMS factor or interaction between factors (Fig. 5b).

### Evaluation of protein expression in cortical samples

Quantification of 5HT2AR immunoreactivity revealed two bands corresponding to molecular weights ~75 kDa and ~60 kDa, as previously reported (Fig. 5c and S1). An increase of the density of 5HTR2A 75 kDa band was observed after administration of two doses of psilocybin, both in the control and CUMS animals (F_Psil_(1,27) = 8.124, *p* = 0.008) (Fig. 5d). A trend towards increase of 5HT2AR 60 kDa band was also observed after psilocybin treatment (F_Psil_(1,28) = 3.607, *p* = 0.07) (Fig. 5e). Positive correlation of the immunodensity of the two bands was also confirmed (r = 0.64; *p* < 0.0001) (Figure S5). Accordingly, 5HT2AR total density showed a significant effect for psilocybin treatment both in the control and CUMS animals (F_Psil_(1,28) = 4.99, *p* = 0.033) (Fig. 5f). In contrast, administration of a single dose of psilocybin did not induce alterations in the 5HT2AR expression in non-stressed mice (Figure S6).

The quantification of BDNF immunoreactivity also revealed three bands corresponding to molecular weights ~42 kDa, ~32 kDa and ~14 kDa, as described previously by other authors (Figure S1). Regarding BDNF 42 kDa, CUMS animals showed decreased relative protein expression (F_CUMS_(1,28) = 4.745, *p* < 0.05). However, psilocybin treatment did not cause any impact (Fig. 5g). CUMS animals also showed decreased relative protein expression of BDNF 32 kDa (F_CUMS_(1,28) = 5.857, *p* < 0.05) (Fig. 5h) and BDNF 14 kDa (F_CUMS_(1,28) = 4.48; *p* < 0.05) (Fig. 5i). Yet, psilocybin treatment did not exert effects on the CUMS-induced decrease.

The quantification of SV2A, PSD-95 and spinophilin immunoreactivity revealed no effects of stress or psilocybin treatment, nor significant interaction between factors (Fig. 5j, k, l).

## Discussion

In the present work, we have conducted a wide behavioural characterization of the effects of psilocybin in an animal model of depression based on chronic unpredictable stress exposure, exploring a broad range of distinctive traits of the disease. Different tests were classified into four dimensions: anhedonia, behavioural despair, apathy and anxious phenotype [58]. These behavioural endpoints are models of dimensions described in human symptomatology [53], which have also been defined as good predictors of antidepressant treatment outcome [59, 60]. Additionally, we have assessed potential mechanistic pathways of the drug through the study of 5HT2AR, HPA and various neuroplasticity-related proteins.

Anhedonia is one of the core clinical features of depression. There is significant induction of anhedonic-like behaviour in rodents upon implementation of CUMS protocols [24], and several types of antidepressants have been proven to reverse it [25, 61, 62]. When tested in the SP, anhedonic state exhibited by the CUMS mice during stress protocol was reversed by psilocybin treatment. Other works have carried out related approaches. Hesselgrave et al. also described psilocybin-induced reversion of anhedonia in a chronic multimodal stress model (restraint stress and exposition to strobe lighting and white noise during 14 days), but only the acute effect of the drug (1 mg/kg, i.p.) was evaluated [34]. Cameron et al. identified that a single dose of psilocybin (10 mg/kg, i.p.) restored corticosterone-induced anhedonia in mice [32]. Sekssaoui et al. showed that psilocybin (1 mg/kg, i.p.) reversed anhedonia induced by repeated swimming in mice (10 min daily during 5 consecutive days) [33]. In addition, chronic microdosing with psilocybin in rats (0.05 mg/kg, s.c. during 24 days) or in mice (0.05 mg/kg, i.p. during 6 days) has shown increased resistance to stress-induced anhedonia, when produced by either repeated saline injections or swimming exposure [33, 63]. In the present study, we first demonstrate that two doses of psilocybin (1 mg/kg, i.p.) administered in two consecutive weeks, which resembles the posology followed in the clinical trials with psilocybin [5–7], are able to exert long-lasting anti-anhedonia effect in a translational animal model of depression.

Behavioural despair is commonly assessed by the FST and the TST, which have been described as useful tools to identify susceptibility to depressive-behaviour induction [64]. The immobility is thought to reflect failure of persistence in escape-directed behaviour, or the development of a passive behaviour that disengages the animal from active forms of coping with stressful stimuli [52]. Several antidepressant drugs have shown efficacy to decrease immobility time in naïve animals [65, 66] but also in mice subjected to CUMS [67–69]. In the present study, the behavioural despair evidenced by increased immobility-time in CUMS was rescued in psilocybin-treated group, both in the FST and the TST. In addition, we have discriminated swimming and climbing behaviours in the FST. Notably, psilocybin was able to rescue swimming but not climbing reduction induced by stress. The swimming is believed to reflect the brain serotonergic system in rodents, whereas climbing relies on the noradrenergic system [51, 52, 70]. This fact argues the idea that long-term antidepressant action of psilocybin might imply serotonergic pathways modulation. Conversely, another study reported no effect of psilocybin in the FST in naïve mice [34]. The discrepancy between results may lie in the already existing behavioural impairment evidenced by passive coping behaviour adopted by stressed mice, as well as the treatment posology and timing of tests. In fact, another recent study has reported long-term, sustained antidepressant effects of psilocybin in FST even 3 weeks after drug-administration in unstressed mice [28], or 2 weeks after, in the chronic behavioural despair model [33]. In contrast, other aspects of the depression-like symptomatology induced by stress were not affected by psilocybin. In line with previous literature [71], we observed decreased auto grooming and nest quality in CUMS mice. The behaviours were previously shown to be rescued by chronic fluoxetine treatment [45] but not by psilocybin in our present study.

Regarding anxiety-like behaviour, multiple studies have described anxiolytic properties of different 5HT2AR agonists [36, 72, 73], including psilocybin [28, 31, 33]. According to the present work, while the effect of psilocybin did not reach statistical significance in the individual behavioural tests for anxiety-like behaviours, we found an overall statistically significant decrease in anxiety-like behaviour in both non-stressed and stressed mice when using the z-score that integrates the behavioural outcomes of the individual tests (see Figure S3). This supports the idea that both disease animal models and naïve mice are suitable for preclinical evaluation of the anxiolytic effect induced by psychedelics. It is noteworthy that, in order to circumvent the occurrence of false negatives, it appears expedient to conduct a series of behavioural tests to assess the anxiety dimension of the disease.

There is a well-established link between chronic stress, HPA dysfunction and depression, evidenced by increased levels of cortisol in plasma, saliva and urine, increased size and activity of pituitary and adrenal glands in patients [74]. Altered expression and functional changes in the GR have also been reported in neuropathological studies of *postmortem* human brains [75]. Additionally, pre-clinical models of decreased GR expression have exhibited depressive-like behaviour [75]. Likewise, alterations in GR mRNA expression have been found in various brain regions of CUMS models [23]. Although we did not find differences in GR mRNA expression in CUMS, we have demonstrated impaired function of the HPA, as stressed mice showed pronounced adrenal gland hypertrophy, which would likely be accompanied by a sustained elevation in plasma corticosterone concentration. Psilocybin treatment caused a selective increase in relative expression of GR in brain cortex of CUMS mice, but not in non-stressed group. These results suggest that psilocybin modifies gene expression of the GR only in the presence of an underlying HPA dysregulation caused by chronic stress. In agreement, antidepressant treatment has been previously linked to the re-establishment of normal HPA function through increased GR expression [74], and this phenomenon seems an indicator of positive long-term therapeutic outcomes. Consistently, CUMS animals also presented alterations in the bodyweight, food intake and fat storage in response to chronic stress, in line with previous studies in mice [76] and rats [77]. Nevertheless, psilocybin did not modify bodyweight, feeding behaviour or fat storage, coherent with data from a study in a mouse model of obesity [78].

The ability of psychedelics to promote neuroplasticity has been proposed as a potential mechanism of psilocybin to exert antidepressant effects [15, 26]. We have assessed potential changes in neuroplasticity-related protein expression in brain cortex. Notably, a decrease in BDNF expression in the cortex of CUMS animals was observed, as it has been previously reported in chronic stress animal models [23, 79] and in the PFC of individuals with depression [80, 81]. Moreover, such neurotrophin seems to be increased in patients after antidepressant treatment [82, 83]. In the present study, no effect of psilocybin was observed on cortical expression of BDNF. Previous studies have also reported transient increases of BDNF following psychedelic administration in plasma of healthy subjects [84] or in rodent brain [15, 78, 85]. Although the predominant mode of BDNF secretion is from presynaptic sites, BDNF can be synthesized and secreted from different components of the synapse including astrocytes, microglia and post-synaptic dendrites [86, 87]. Thus, it is feasible to speculate that psychedelic-induced neuroplastic effect could be mediated by the selective increase of BDNF from precise subcellular localizations [88], and a more specific look into local production of BDNF in the dendritic compartment may shed light into their mechanism of action. Moreover, discrepancy between different studies could be due to the desynchronised timing between tissue harvest (14 days after the last dose in the present work) and the “window of neuroplasticity” opened by psilocybin [89].

Additionally, we measured the 12-transmembrane domain glycoprotein SV2A. This protein is expressed in synaptic vesicles throughout the brain and is considered to reflect presynaptic density [90]. One study performed in pigs has reported increases in cortical SV2A seven days after one single intravenous administration of psilocybin [91]. Kiilerich et al., have also measured increases in SV2A levels in the paraventricular thalamic nucleus after repeated low doses of psilocybin in mice [63]. Unfortunately, the present work was not able to replicate such results, which could be due to differences in the methodological approach, species, region-specificity or experiment timing. Other neuroplasticity-related markers were also measured. Little evidence exists on the regulation of postsynaptic density protein PSD-95, and while one study showed a rapid increase in the cortical *Psd95* gene expression in a region-specific manner after psilocybin (8 mg/kg i.p.) in rats [92], another did not (1 mg/kg i.p.) [93]. Current data does not show changes in the PSD-95 protein expression in psilocybin-treated mice. Finally, post-synaptic actin-binding protein spinophilin was evaluated. It is known to play a major role in spine formation and function, and to regulate cytoskeletal function [94]. Evidence suggests that chronic antidepressant treatment modulates its gene expression in frontal cortex [95]. However, no changes in the cortical protein expression derived from stress or after treatment were found in the present work.

Although the 5HT2AR desensitization upon a high dose of psychedelic administration has been described [96, 97], few studies have assessed 5HT2AR mRNA and protein expression after psilocybin treatment. Unexpectedly, the cortical 5HT2AR expression was increased in both psilocybin-treated groups, regardless of control or stress condition, even though the antidepressant-like effects of psilocybin were exclusively present in CUMS group. Previous works have shown no changes in the cortical *Htr2a* mRNA 1 day or 7 days after single administration of psilocybin in pigs [98], or a transient decrease and return to baseline few hours following DOI in rats [99]. To the best of our knowledge, this is the first work to report a long-term effect in 5HT2AR in brain cortex after two systemic administrations of psilocybin. In addition, detection of two bands in Western blot allows to speculate that different subpopulations of 5HT2AR may be detected in total homogenates of cortical samples. Although the nature of this modification remains unknown, it could be aimed at correct 5HT2AR folding, targeting to the membrane or its activity at the membrane (eg. glycosylation). Alternatively, it may be involved in addressing 5HT2AR to organelle membranes (e.g., lipidation as palmitoylation) or even in trafficking the internalized receptor to downstream degradation processes (e.g., ubiquitination, SUMOylation). In accordance with higher 5HT2AR expression, we also observed an increase in the HTR at the second administration of psilocybin in control and CUMS groups; confirming this phenomenon as a stress-independent effect. This phenomenon is not in line with the tolerance developed after repeated administrations of the psychedelic DOI (consecutive daily doses) [97, 100]. Interestingly, previous works have reported that challenge DOI doses, when administered in non-consecutive days, are able to produce significantly greater effects relative to their respective first-injection control values [101–103], as reported for psilocybin in the present work. Higher in vivo sensitivity to psilocybin, along with an enhancement of cortical 5HT2AR density could reflect a long-term over-compensatory mechanism after initial receptor desensitization. A more exhaustive assessment of the receptor status at various time points after single and booster dose of psilocybin could provide clues into the mechanism driving this action and should be taken into consideration for future studies. It is crucial to ascertain whether these effects are exclusive to psilocybin or can be extrapolated to other tryptamine-derived psychedelics or also to phenethylamine- and ergoline-derived psychedelic drugs. In this context, previous studies have demonstrated the existence of cross-interactions between the psychedelic DOI (phenethylamine) and lysergic acid diethylamide (LSD, an ergoline) [97]. This information is relevant to the safety of future treatments with psychedelic drugs with different pharmacological profiles. The present study likewise indicates the value of integrating positron emission tomography (PET) techniques with 5HT2AR selective ligands such as [^11^C]Cimbi-36 into clinical trials to evaluate the acute and long-term consequences of pharmacological interventions with psychedelics, in line with previous recommendations [104].

### Novelty and limitations of the study

The present study builds upon the findings of a previous study in which a dose-response analysis was conducted to evaluate the acute effects of psilocybin [14]. Moreover, this study mimicked a multi-administration scheme already evaluated in different clinical trials [5–7, 10]. While several studies have assessed the antidepressant and anxiolytic effects of psilocybin in animals [105], this is the first to examine its long-term therapeutic effects in a pre-clinical model based on chronic and unpredictable stress—one of the most translational models for investigating depression-associated symptoms.

The development of the animal model required prior optimization of the protocol to ensure the emergence of a behavioural phenotype exhibiting depression-associated symptoms across an extensive battery of behavioural tests. The effect of psilocybin was then studied in this pathological phenotype. Taken together, these factors provide construct, content, and predictive validity to the present study, highlighting its novelty and relevance, and contributing to the reproducibility of the results obtained in preclinical trials.

The study has also several limitations, including sex bias and lack of data in female mice. Unfortunately, preclinical studies evaluating antidepressant-like effects in females are scarce, despite depression being twice as common in women as in men. Regrettably, the effect of sex as a variable could not be included in the present study, but the authors acknowledge and forcefully recommend the use of both sexes in basic experimental research, especially with regard to characterisation of antidepressants. Another important drawback, as already mentioned, is the extended time interval between the last administration of psilocybin and tissue harvest, due to the need to perform the in vivo assessment. It is reasonable to think that a closer time point would be more suitable for the evaluation of certain molecular pathways targeted by the drug, to unfold the molecular basis of the therapeutic action of psilocybin.

## Conclusions

Overall, our results provide new insight into the effects of psilocybin in a well-validated, translational model of depression. Chronic stress model of depression exhibited disrupted behaviour relative to different dimensions of depressive-like and anxiety-like symptomatology. Two doses of psilocybin were able to rescue impairments in depressive phenotype, and to induce anxiolytic-like effects. Additionally, an increase in sensitivity to psilocybin-induced HTR was observed following booster dose. Altogether, these results highlight that the use of translational animal models contributes to the understanding of the largely debated therapeutic effect and mechanism of action of psychedelics. This preclinical model can be useful in order to implement effective and feasible treatment models of psychedelic-assisted therapies for depression. The results of this study indicate a need for further evaluation of the therapeutic potential of psilocybin in treating anxiety symptoms associated with depression in future clinical trials. Further studies are warranted to address mechanistic aspects of the antidepressant/anxiolytic effect of psilocybin, including sex bias.

## Supplementary information

Supplemental material

## Data Availability

The data sets generated for this study are available on request to the corresponding authors.
