# Supplementary material for: Evaluation of behavioural and neurochemical effects of psilocybin in mice subjected to chronic unpredictable mild stress
Source: Transl Psychiatry. 2025 Jun 14;15:201. doi: 10.1038/s41398-025-03421-4 (PMC12167372; doi:10.1038/s41398-025-03421-4)
Supplement: Supplementary file 1 — Supplemental material [file 41398_2025_3421_MOESM1_ESM.docx]

**Table S1.** CUMS protocol: stressful stimuli, duration, details and intensity according to the severity point system. Additionally, CUMS mice were individually housed for the duration of the experimental protocol. D: dark (active) phase of the cycle. L: light (inactive) phase of the cycle.

| ***Stressor*** | ***Description*** | ***Duration*** | ***Phase of the cycle*** | ***Intensity*** |
| --- | --- | --- | --- | --- |
| **Cage tilting** | Cages were tilted sideways 45º | 12 h | D | 1 |
| **Overcrowding** | 8 animals were placed in an individual cage | 2 h | L | 1 |
| **Food/water deprivation** | Food and water were removed | 8 h | D | 1 |
| **Continuous light** | Lights were kept on for on entire sleep cycle | 24 h | L + D | 2 |
| **Light pulses** | Lights were switched on and off every 20 min | 12 h | D | 2 |
| **Predator odour** | Sawdust was removed and replaced with sawdust from rats’ cages | 2 h | L | 2 |
| **Alarm clock** | An alarm clock at 85 dB was activated at random times | 10 min | L + D | 2 |
| **Wet bedding** | 200 mL of water were poured over 400 mL of sawdust | 12 h | D | 2 |
| **No bedding** | Sawdust was removed | 12 h | D | 2 |
| **White noise** | An untuned radio was set at 85 dB | 4 h | L | 2 |
| **Cold exposure** | Cages were introduced in a chamber at 4 ºC | 1 h | L | 3 |
| **Heat exposure** | A heat source was placed in the rack. Temperature did not exceed 40 ºC | 2 h | L | 3 |
| **Water bath** | Sawdust was removed and replaced with warm (23 ºC) water (2 cm-high) | 2 h | L | 3 |
| **Stroboscopic lights** | Flashing lights were applied | 4 h | L | 3 |
| **Restraint** | Mice were introduced in ventilated plastic falcons (12 x 3 cm) | 2 h | L | 3 |


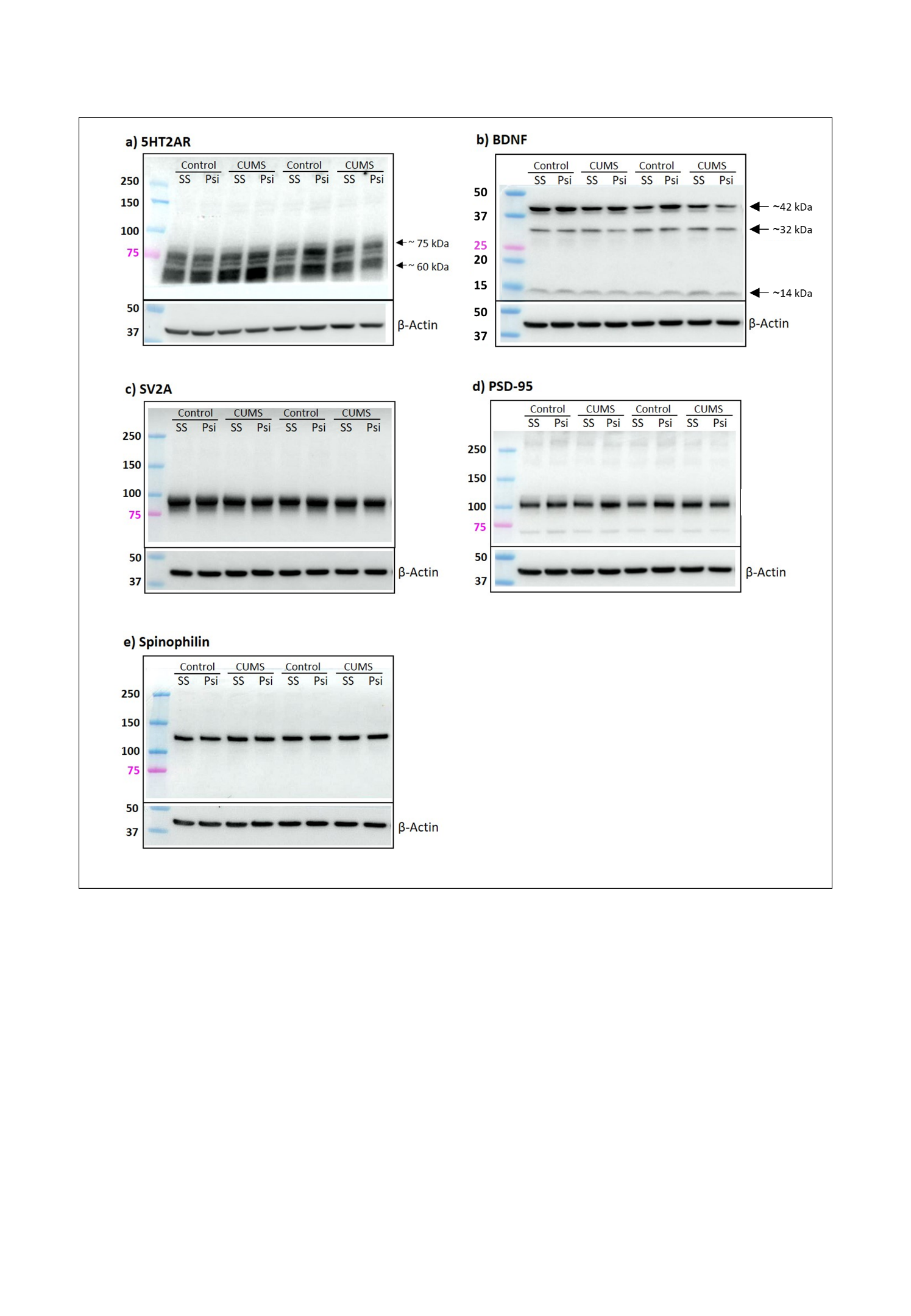


**Figure S1.** Representative Western blot images of the expression of different proteins in brain cortex. Expression of control and CUMS mice are shown. Images represent immunoblots of: 5HT2AR (**a**), BDNF (**b**), SV2A (**c**), PSD-95 (**d**) and Spinophilin (**e**). Each protein blot is accompanied by the respective β-actin blot.

|  |  |  | |  | **Two-way ANOVA** | | |
| --- | --- | --- | --- | --- | --- | --- | --- |
| **Parameter** | **Theoretical “n”** | **Actual “n”** | **Animals removed** | | | **Theoretical degrees of freedom in two-way ANOVA** | **Actual degrees of freedom in two-way ANOVA** |
| HTR 1^st^ dose | 48 | 46 | 1 outlier (Grubb’s test); 1 wrong video recording | | | F(1,44) | F(1,42) |
| HTR 2^nd^ dose | 48 | 47 | 1 wrong video recording | | | F(1,44) | F(1,43) |
| SP week 6 (after treatment) | 64 | 51 | 12 methodological problems with sucrose or water bottles (leaking); 1 mouse deceased | | | F(1,60) | F(1,47) |
| TST immobility | 64 | 50 | 3 outliers (Grubb’s test); 10 mice climbed up the tail; 1 mouse deceased | | | F(1,60) | F(1,46) |
| FST immobility | 64 | 63 | 1 mouse deceased | | | F(1,60) | F(1,59) |
| FST swimming | 64 | 63 | 1 mouse deceased | | | F(1,60) | F(1,59) |
| FST climbing | 64 | 63 | 1 mouse deceased | | | F(1,60) | F(1,59) |
| EPM open arm entries % | 64 | 61 | 1 outlier (Grubb’s test); 1 wrong video recording; 1 mouse deceased | | | F(1,60) | F(1,57) |
| EPM closed arm time | 64 | 61 | 1 outlier (Grubb’s test); 1 wrong video recording; 1 mouse deceased | | | F(1,60) | F(1,57) |
| EPM latency to closed arms | 64 | 59 | 4 outliers (Grubb’s test); 1 mouse deceased | | | F(1,60) | F(1,55) |
| OFT distance | 64 | 63 | 1 mouse deceased | | | F(1,60) | F(1,59) |
| OFT time in centre | 64 | 62 | 1 outlier (Grubb’s test); 1 mouse deceased | | | F(1,60) | F(1,58) |
| NSFT latency to feed | 64 | 59 | 4 outliers (Grubb’s test); 1 mouse deceased | | | F(1,60) | F(1,55) |
| Bodyweight gain | 64 | 63 | 1 mouse deceased | | | F(1,60) | F(1,59) |
| Food intake | 64 | 62 | 1 methodological problem; 1 mouse deceased | | | F(1,60) | F(1,58) |
| WAT | 64 | 62 | 1 outlier (Grubb’s test); 1 mouse deceased | | | F(1,60) | F(1,58) |
| BAT | 64 | 62 | 1 outlier (Grubb’s test); 1 mouse deceased | | | F(1,60) | F(1,58) |
| Adrenal glands | 64 | 61 | 1 outlier (Grubb’s test); 1 methodological problem during harvest; 1 mouse deceased | | | F(1,60) | F(1,57) |
| Depression index | 64 | 63 | 1 mouse deceased | | | F(1,60) | F(1,59) |
| Anxiety index | 64 | 62 | 1 outlier (Grubb’s test); 1 mouse deceased | | | F(1,60) | F(1,58) |
| Physiological signs of stress | 64 | 62 | 1 outlier (Grubb’s test); 1 mouse deceased | | | F(1,60) | F(1,58) |
| *Nr3c1* expression | 32 | 28 | 2 outliers (Grubb’s test); 2 methodological problems when pipetting | | | F(1,28) | F(1,24) |
| *Htr2a* expression | 32 | 31 | 1 outlier (Grubb’s test) | | | F(1,28) | F(1,27) |
| 5-HT2AR 75 kDa | 32 | 31 | 1 outlier (Grubb’s test) | | | F(1,28) | F(1,27) |
| 5-HT2AR 60 kDa | 32 | 32 | - | | | F(1,28) | F(1,28) |
| 5-HT2AR total | 32 | 32 | - | | | F(1,28) | F(1,28) |
| BDNF 42 kDa | 32 | 32 | - | | | F(1,28) | F(1,28) |
| BDNF 32 kDa | 32 | 32 | - | | | F(1,28) | F(1,28) |
| BDNF total | 32 | 32 | - | | | F(1,28) | F(1,28) |
| SV2A | 32 | 32 | - | | | F(1,28) | F(1,28) |
| PSD-95 | 32 | 31 | 1 outlier (Grubb’s test) | | | F(1,28) | F(1,27) |
| Spinophilin | 32 | 32 | - | | | F(1,28) | F(1,28) |

**Table S2.** Theoretical and final degrees of freedom obtained in two-way ANOVA

**Table S3.** Results of Two-way ANOVA analyses (F, df and p values). Results in **bold** represent statistically significant values.

|  | **Two-way ANOVA** | | |
| --- | --- | --- | --- |
| **Parameter** | **CUMS** | **Psilocybin** | **Interaction** |
| HTR 1^st^ dose | F_(1,42)_=1.37; p=0.25 | **F_(1,42)_=182.60; p<0.0001** | F_(1,42)_=2.51; p=0.12 |
| HTR 2^nd^ dose | F_(1,43)_=0.84; p=0.36 | **F_(1,43)_=405.20; p<0.0001** | F_(1,43)_=1.49; p=0.23 |
| SP week 6 (after treatment) | F_(1,47)_=1.62; p=0.21 | F_(1,47)_=0.03; p=0.85 | **F_(1,47)_=9.92; p<0.01** |
| TST immobility | F_(1,46)_=2.76; p=0.10 | **F_(1,46)_=9.91; p<0.01** | **F_(1,46)_=9.05; p<0.01** |
| FST immobility | **F_(1,59)_=19.74; p<0.0001** | **F_(1,59)_= 9.02; p<0.01** | **F_(1,59)_= 4.45; p<0.05** |
| FST swimming | **F_(1,59)_=4.74; p<0.05** | **F_(1,59)_=5.24; p<0.05** | **F_(1,59)_= 11.67; p<0.01** |
| FST climbing | **F_(1,59)_=10.98; p<0.01** | F_(1,59)_=1.73; p=0.19 | F_(1,59)_=1.27; p=0.26 |
| EPM open arm entries % | **F_(1,57)_=6.50; p<0.05** | F_(1,57)_=1.83; p=0.18 | F_(1,57)_=0.08; p=0.79 |
| EPM closed arm time | **F_(1,57)_=14.91; p<0.001** | F_(1,57)_=0.42; p=0.52 | F_(1,57)_=0.13; p=0.72 |
| EPM latency to closed arms | **F_(1,55)_=9.37; p<0.01** | F_(1,55)_=1.79; p=0.19 | F_(1,55)_=0.08; p=0.78 |
| OFT distance | **F_(1,59)_=5.21; p<0.05** | **F_(1,59)_=15.40; p<0.001** | F_(1,59)_=0.01; p=0.92 |
| OFT time in centre | **F_(1,58)_=7.38; p<0.01** | F_(1,58)_=1.98; p=0.17 | F_(1,58)_=0.10; p=0.75 |
| NSFT latency to feed | **F_(1,55)_=9.35; p<0.01** | F_(1,55)_=0.84; p=0.36 | F_(1,55)_=0.02; p=0.89 |
| Bodyweight gain | **F_(1,59)_=140.40; p<0.0001** | F_(1,59)_=0.08; p=0.78 | F_(1,59)_=0.70; p=0.41 |
| Food intake | **F_(1,58)_=134.20; p<0.0001** | F_(1,58)_=0.34; p=0.56 | F_(1,58)_=0.40; p=0.53 |
| WAT | **F_(1,58)_=25.43; p<0.0001** | F_(1,58)_=0.94; p=0.34 | F_(1,58)_=0.18; p=0.67 |
| BAT | **F_(1,58)_=25.03; p<0.0001** | F_(1,58)_=0.02; p=0.89 | F_(1,58)_=0.49; p=0.49 |
| Adrenal glands | **F_(1,57)_=193.00; p<0.0001** | F_(1,57)_=0.002; p=0.99 | F_(1,57)_=0.55; p=0.46 |
| Depression index | **F_(1,59)_=13.18; p<0.001** | **F_(1,59)_=8.42; p<0.01** | **F_(1,59)_=24.27; p<0.0001** |
| Anxiety index | **F_(1,58)_=18.26; p<0.0001** | **F_(1,58)_=4.02; p<0.05** | F_(1,58)_=0.27; p=0.61 |
| Physiological signs of stress | **F_(1,58)_=446.70; p<0.0001** | F_(1,58)_=0.05; p=0.83 | F_(1,58)_=0.25; p=0.61 |
| *Nr3c1* expression | **F_(1,24)_=8.76; p<0.01** | F_(1,24)_=0.32; p=0.58 | **F_(1,24)_=20.66; p=0.0001** |
| *Htr2a* expression | F_(1,27)_=0.03; p=0.86 | **F_(1,27)_=5.55; p<0.05** | F_(1,27)_=0.07; p=0.79 |
| 5-HT2AR 75 kDa | F_(1,27)_=1.74; p=0.20 | **F_(1,27)_=8.12; p<0.01** | F_(1,27)_=0.11; p=0.74 |
| 5-HT2AR 60 kDa | F_(1,28)_=0.95; p=0.34 | F_(1,28)_=3.61; p=0.07 | F_(1,28)_=0.51; p=0.48 |
| 5-HT2AR total | F_(1,28)_=0.20; p=0.66 | **F_(1,28)_=4.99; p<0.05** | F_(1,28)_=0.76; p=0.39 |
| BDNF 42 kDa | **F_(1,28)_=4.75; p<0.05** | F_(1,28)_=0.12; p=0.74 | F_(1,28)_=0.11; p=0.74 |
| BDNF 32 kDa | **F_(1,28)_=5.86; p<0.05** | F_(1,28)_=1.70; p=0.20 | F_(1,28)_=0.23; p=0.64 |
| BDNF 14 kDa | **F_(1,28)_=4.48; p<0.05** | F_(1,28)_=0.06; p=0.81 | F_(1,28)_=0.01; p=0.94 |
| SV2A | F_(1,28)_=0.32; p=0.58 | F_(1,28)_=0.17; p=0.68 | F_(1,28)_=0.06; p=0.82 |
| PSD-95 | F_(1,27)_=0.61; p=0.44 | F_(1,27)_=0.04; p=0.84 | F_(1,27)_=0.77; p=0.39 |
| Spinophilin | F_(1,28)_=0.09; p=0.77 | F_(1,28)_=0.66; p=0.42 | F_(1,28)_=0.15; p=0.70 |

**Table S4**. Summary of results of behavioural and physiological parameters for each experimental group, expressed as means ± SEM. Results in **bold** indicate significant effect of psilocybin factor in two-way ANOVA. Underlined results indicate significant effect of CUMS factor in two-way ANOVA or in unpaired t-test (SP week 4). In the case of significant interaction between factors, only *post hoc* analysis is represented as “ * ” for control-saline vs CUMS-saline, and “ ^#^ ” for CUMS-saline vs CUMS-psilocybin.

|  | **Experimental group and treatment** | | | |
| --- | --- | --- | --- | --- |
| **Behavioural parameter** | **Control saline** | **Control psilocybin** | **CUMS saline** | **CUMS psilocybin** |
| SP week 4 (before treatment) (%) | 83.41 ± 1.35 | | 71.95 ± 2.47 | |
| SP week 6 (after treatment) (%) | 85.72 ± 1.93 | 79.16 ± 2.36 | 75.93 ± 2.51* | 83.32 ± 1.22^#^ |
| TST immobility (s) | 75.99 ± 6.14 | 74.99 ± 7.43 | 109.33 ± 6.15* | 65.38 ± 7.23^#^ |
| FST immobility (s) | 178.30 ± 5.66 | 174.30 ± 4.27 | 207.64 ± 2.32* | 184.74 ± 5.00^#^ |
| FST swimming (s) | 53.42 ± 2.96 | 49.02 ± 4.91 | 31.60 ± 2.16* | 53.85 ± 4.95^#^ |
| FST climbing (s) | 8.28 ± 3.75 | 16.68 ± 5.57 | 0.76 ± 0.45 | 1.41 ± 0.72 |
| EPM open arm entries (%) | 31.29 ± 1.72 | 33.85 ± 2.44 | 24.58 ± 2.69 | 28.45 ± 2.38 |
| EPM closed arm time (s) | 159.09 ± 10.33 | 148.96 ± 12.51 | 194.25 ± 8.89 | 191.42 ± 7.41 |
| EPM latency to closed arms (s) | 5.99 ± 1.55 | 8.35 ± 2.13 | 1.95 ± 0.65 | 3.48 ± 0.96 |
| OFT distance (cm) | 1337.82 ± 97.46 | **1751.10 ± 111.69** | 1582.38 ± 95.25 | **1975.29 ± 105.67** |
| OFT time in centre (s) | 24.26 ± 5.68 | 29.70 ± 7.03 | 9.01 ± 1.32 | 17.68 ± 3.55 |
| NSFT latency to feed (s) | 283.20 ± 31.92 | 248.94 ± 25.34 | 378.00 ± 29.99 | 352.87 ± 40.46 |
| NBT score | 4.63 ± 0.13 | 4.50 ± 0.16 | 2.06 ± 0.23 | 1.60 ± 0.19 |
| Coat state score | 0.09 ± 0.05 | 0.16 ± 0.06 | 2.13 ± 0.18 | 2.13 ± 0.20 |
| Change of bodyweight (%) | 32.82 ± 1.08 | 33.59 ± 1.35 | 17.56 ± 1.44 | 16.00 ± 1.64 |
| Food intake (g/g) | 0.11 ± 0.002 | 0.11 ± 0.004 | 0.16 ± 0.01 | 0.16 ± 0.01 |
| WAT weight (mg/g) | 15.92 ± 0.75 | 15.01 ± 0.48 | 12.35 ± 0.66 | 12.00 ± 0.68 |
| BAT weight (mg/g) | 5.05 ± 0.26 | 4.88 ± 0.30 | 6.32 ± 0.26 | 6.57 ± 0.35 |
| Adrenal gland weight (mg/g) | 0.22 ± 0.01 | 0.23 ± 0.01 | 0.37 ± 0.01 | 0.36 ± 0.01 |

**Table S5**. Summary of results of individual and global z-scores and indexes for each experimental group, expressed as means ± SEM. Results in **bold** indicate significant effect of psilocybin factor in two-way ANOVA. Underlined results indicate significant effect of CUMS factor in two-way ANOVA. In the case of significant interaction between factors, only *post hoc* analysis is represented as “ * ” for control-saline vs CUMS-saline, and “ ^#^ ” for CUMS-saline vs CUMS-psilocybin.

|  | **Experimental group and treatment** | | | |
| --- | --- | --- | --- | --- |
| **Z-score** | **Control saline** | **Control psilocybin** | **CUMS saline** | **CUMS psilocybin** |
| FST immobility | 0.00 ± 0.25 | -0.18 ± 0.19 | 1.84 ± 0.18* | -0.04 ± 0.42^#^ |
| FST swimming | 0.00 ± 0.25 | 0.37 ± 0.42 | 1.30 ± 0.10* | 0.28 ± 0.22 |
| FST climbing | 0.00 ± 0.25 | -0.56 ± 0.37 | 0.50 ± 0.03 | 0.46 ± 0.05 |
| FST global | 0.00 ± 0.22 | -0.12 ± 0.17 | 1.21 ± 0.10* | 0.24 ± 0.21^#^ |
| TST immobility | 0.00 ± 0.26 | -0.04 ± 0.31 | 1.40 ± 0.26* | -0.45 ± 0.30^#^ |
| Behavioural despair | -0.07 ± 0.22 | -0.09 ± 0.17 | 1.23 ± 0.10* | 0.03 ± 0.20^#^ |
| SP = Anhedonia | 0.00 ± 0.27 | 0.91 ± 0.33 | 1.36 ± 0.35* | 0.33 ± 0.17 |
| ***DEPRESSION INDEX*** | -0.02 ± 0.16 | 0.31 ± 0.15 | 1.37 ± 0.17* | 0.10 ± 0.17^#^ |
| EPM closed arms time | 0.00 ± 0.27 | -0.26 ± 0.32 | 0.91 ± 0.23 | 0.84 ± 0.19 |
| EPM latency to closed arms | 0.00 ± 0.30 | -0.13 ± 0.24 | 0.66 ± 0.11 | 0.41 ± 0.16 |
| EPM open arms time | 0.00 ± 0.27 | 0.29 ± 0.20 | 0.75 ± 0.17 | 0.21 ± 0.22 |
| EPM open arm entries | 0.00 ± 0.27 | -0.40 ± 0.38 | 1.04 ± 0.42 | 0.44 ± 0.37 |
| EPM global | 0.00 ± 0.22 | -0.09 ± 0.27 | 0.91 ± 0.25 | 0.50 ± 0.23 |
| OFT time in centre | 0.00 ± 0.25 | -0.24 ± 0.31 | 0.67 ± 0.06 | 0.29 ± 0.16 |
| OFT distance ratio (DR) | 0.02 ± 1.00 | -1.76 ± 1.14 | 1.35 ± 0.75 | 0.44 ± 0.94 |
| OFT global | 0.01 ± 0.60 | -1.00 ± 0.71 | 1.01 ± 0.39 | 0.36 ± 0.54 |
| NSFT latency to feed | 0.00 ± 0.26 | -0.28 ± 0.21 | 0.77 ± 0.24 | 0.56 ± 0.33 |
| ***ANXIETY INDEX*** | 0.00 ± 0.35 | **-0.54 ± 0.40** | 1.58 ± 0.29 | **0.72 ± 0.29** |
| Bodyweight gain | 0.00 ± 0.25 | -0.18 ± 0.27 | 3.99 ± 0.30 | 4.33 ± 0.39 |
| Food intake | 0.00 ± 0.22 | -0.08 ± 0.33 | 3.20 ± 0.47 | 3.85 ± 0.47 |
| Adrenal gland weight | 0.00 ± 0.25 | 0.09 ± 0.37 | 5.03 ± 0.39 | 4.77 ± 0.41 |
| ***PHYSIOLOGICAL SIGNS OF STRESS*** | 0.00 ± 0.14 | -0.06 ± 0.21 | 4.08 ± 0.21 | 4.22 ± 0.22 |


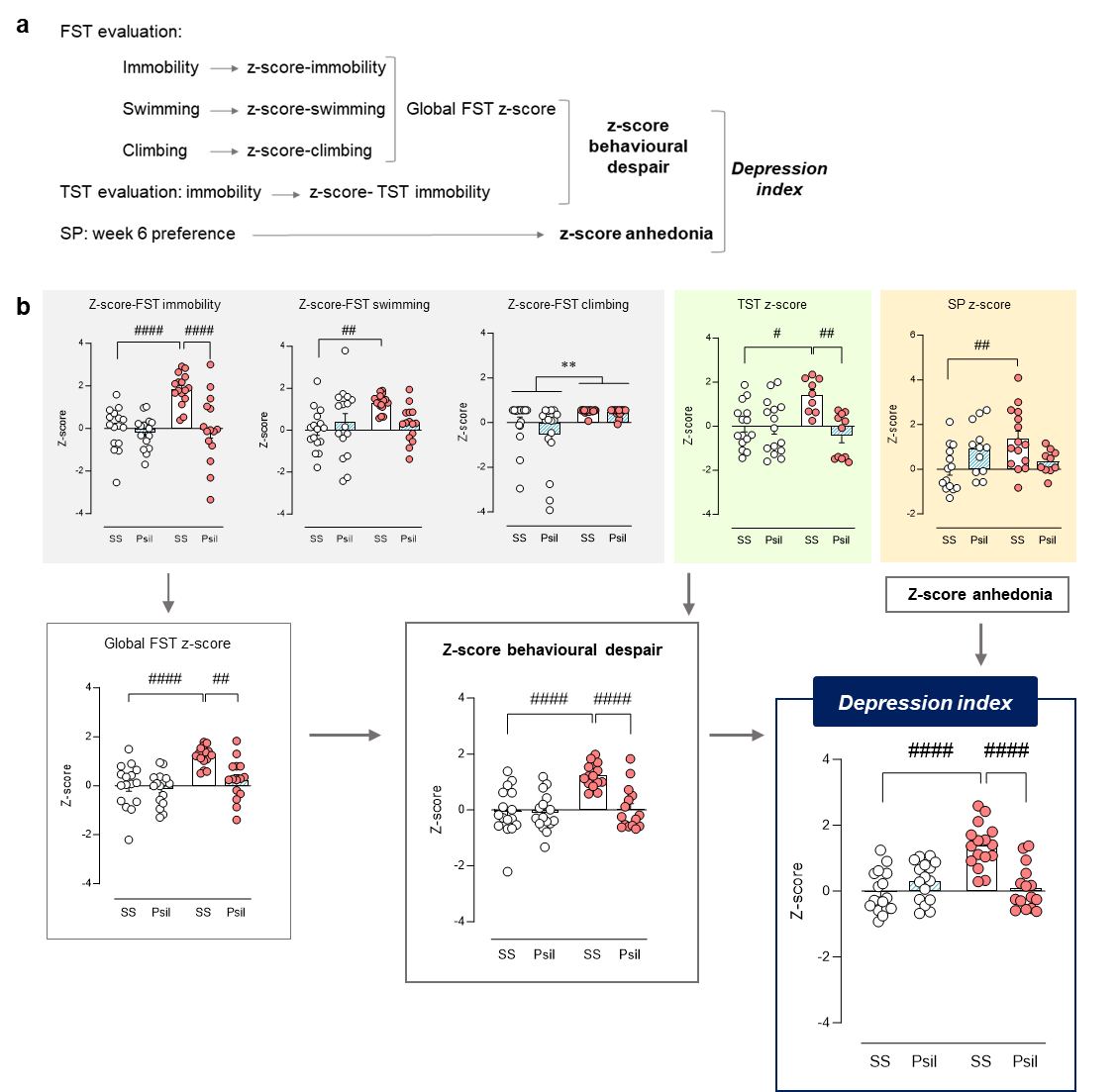


**Figure S2.** (**a**) Flow diagram of depression index calculation, from individual parameter evaluation in behavioural tests, to z-scores of behavioural despair and anhedonia. (**b**) Z-scores for individual parameters evaluated in FST, TST and SP, global z-score for FST, integrated z-scores for behavioural despair and anhedonia. Two-way ANOVA. **p<0.01. Bonferroni *post hoc* test. ^#^p<0.05, ^##^p<0.01, ^###^p<0.001, ^####^p<0.0001.

**
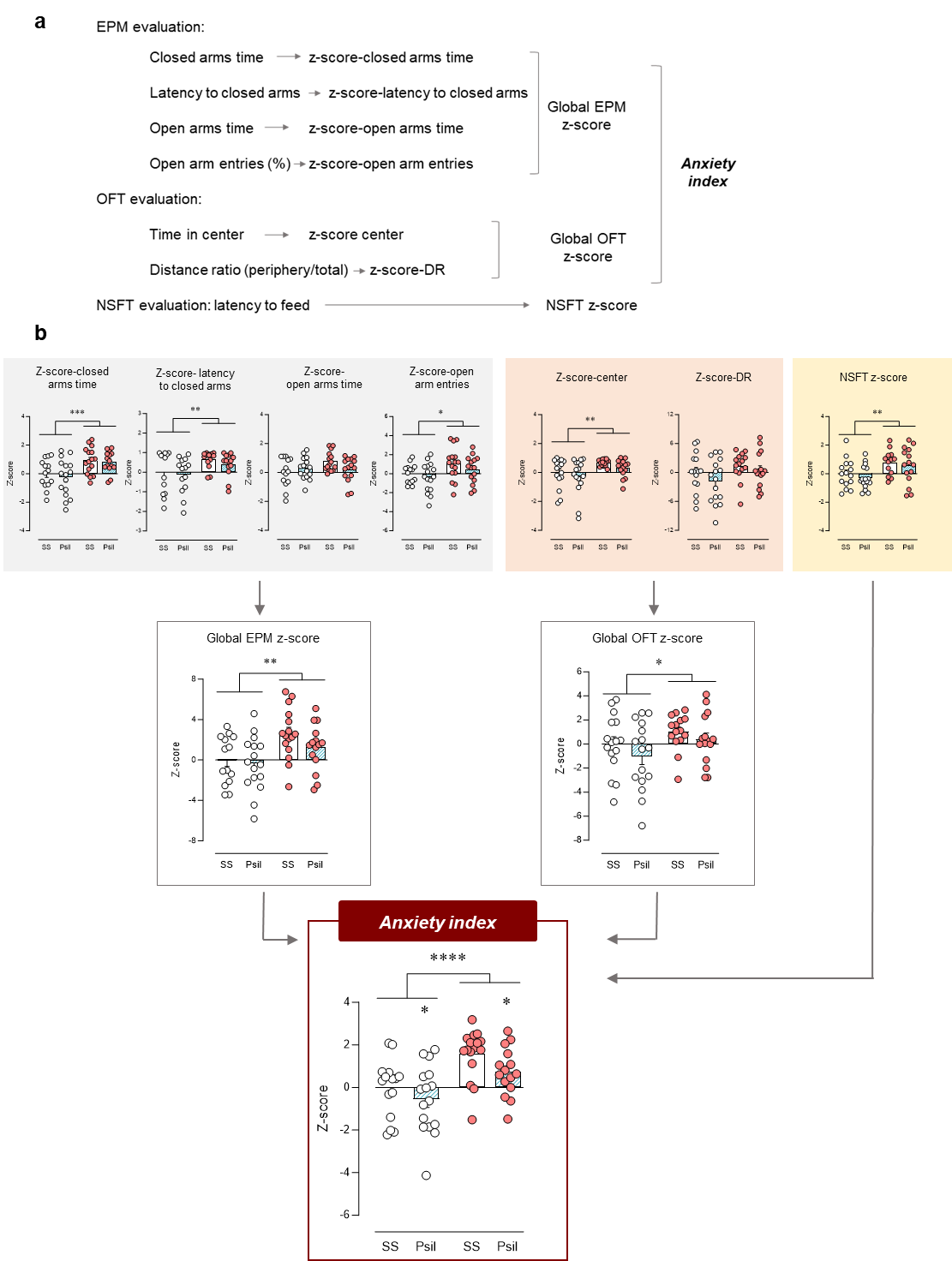
**

**Figure S3.** (**a**) Flow diagram of anxiety index calculation, from individual parameter evaluation, to global z-scores of behavioural tests. (**b**) Z-scores for individual parameters evaluated in EPM, OFT and NSFT and global z-scores for EPM and OFT. Two-way ANOVA. *p<0.05, **p<0.01, ***p<0.001.


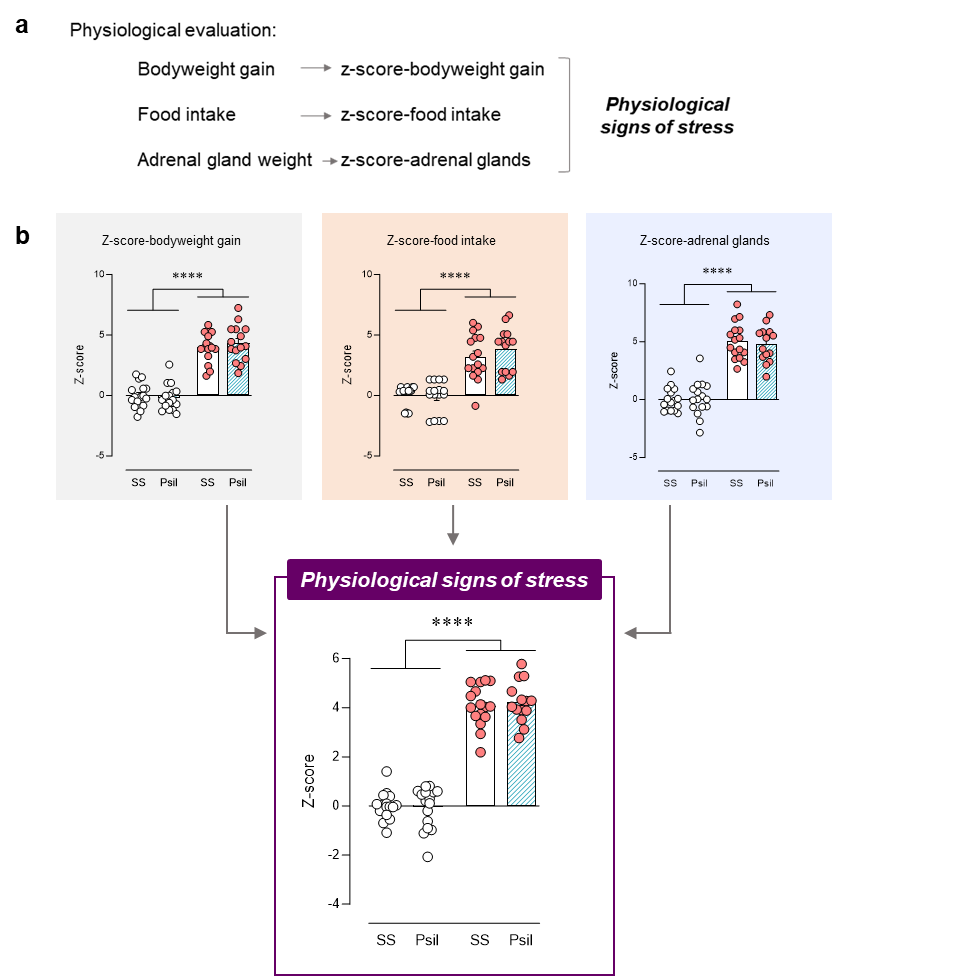


**Figure S4.** (**a**) Flow diagram of calculation of physiological signs of stress, from individual parameter evaluation, to global z-score. (**b**) Z-scores for bodyweight gain, food intake and adrenal gland weight. Two-way ANOVA. ****p<0.0001.


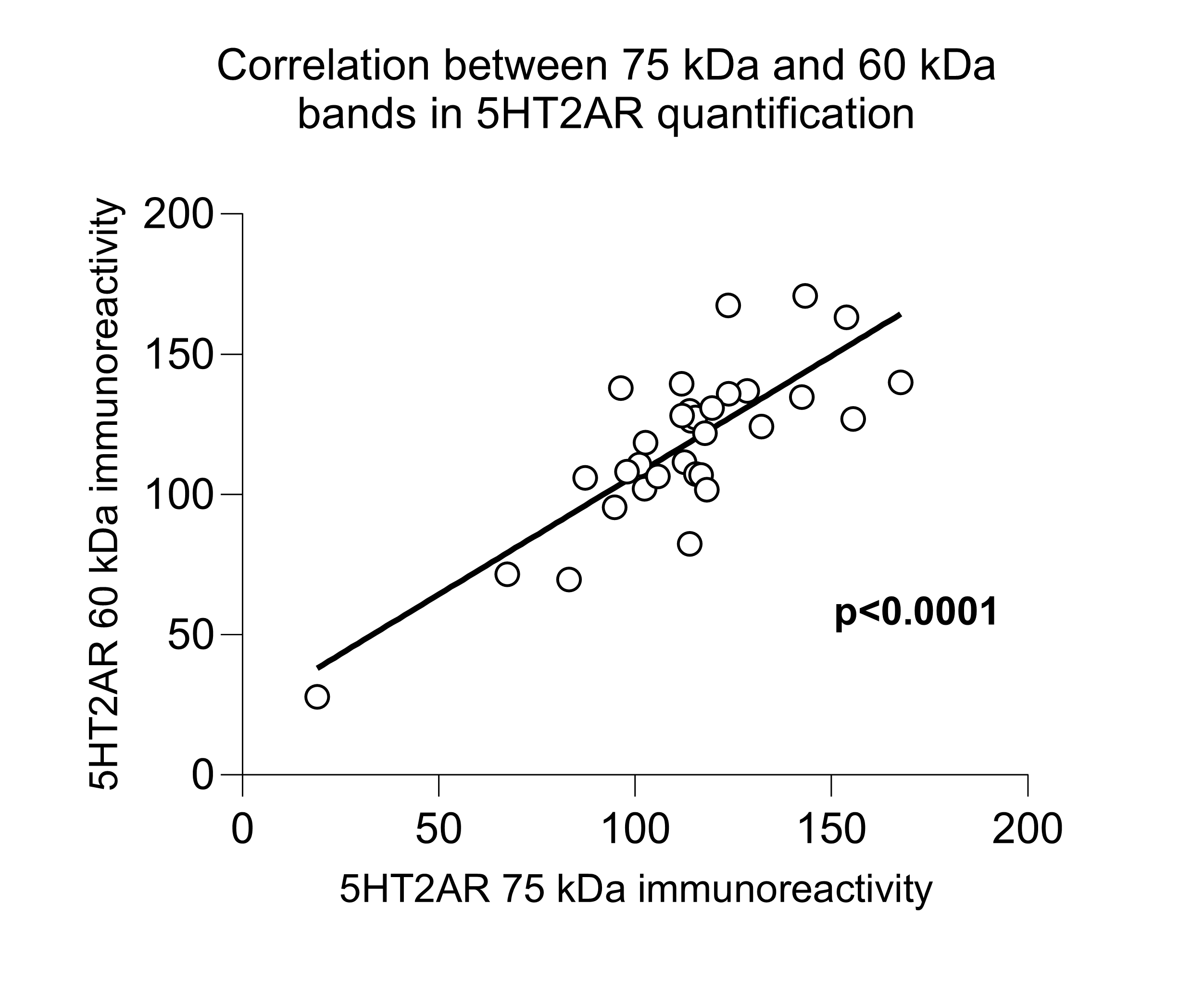


**Figure S5.** Correlation of immunodensity of bands quantified for 5HT2AR.





**Figure S6.** Relative protein expression of 5HT2AR of 75 kDa (**a**), 60 kDa (**b**) and total (**c**) bands after single dose of psilocybin (1 mg/kg i.p.) or saline (5 mL/kg i.p.) administration. Unpaired t-test showed no significant difference between saline and psilocybin treatments.
